# Laboratory Analysis of Backpack Design and Walking Gradient Effects on Gait Kinetics and Kinematics

**DOI:** 10.3390/sports13100350

**Published:** 2025-10-03

**Authors:** Timothy Grigg, Natalia Kabaliuk, Sibi Walter

**Affiliations:** 1 Faculty of Health, University of Canterbury, Christchurch 8041, New Zealand; 2 Department of Mechanical Engineering, University of Canterbury, Christchurch 8041, New Zealand

**Keywords:** lumbar, gait analysis, postural balance, walking

## Abstract

Background: Heavy backpacks are carried by hikers during prolonged trekking trips. A backpack’s design could impact a hiker’s gait kinematics and kinetics. Objective: We aimed to assess the impact of backpack designs on lumbar extension (LE) and centre of pressure (COP) during walking. Methods: Participants (*n* = 8; age = 23 ± 2) attended testing sessions to assess a traditional backpack (TBP) and a balance backpack (BBP) against no backpack (NBP) control while walking on three gradients (flat, 0°; incline, 12°; decline, −12°). Walking tests were conducted on a force plate-embedded treadmill with a motion capture system. Statistical tests assessed the effect of a backpack on LE and COP during carriage. Dunnett’s multiple comparison post hoc test identified significant main effects (5% significance). Results: The observed differences in an individual’s LE and COP across all three gradients were statistically (a = 0.05) significantly less when using a BBP compared to a TBP. Conclusion: Comparative analysis revealed that the BBP’s anterior–posterior loading system closely replicated the gait pattern of unloaded walking across the observed gradients. These findings suggest that hikers using a BBP may exhibit a gait resembling unloaded gait in comparison to a TBP gait.

## 1. Introduction

Outdoor nature walking, also known as hiking, is a popular recreational physical activity [1]; for instance, in 2021, 1.14 million New Zealanders took at least one hike [2]. Hiking involves walking long distances over several days carrying a heavy backpack [3]. Carrying a heavy backpack affects hikers’ walking biomechanics as typical backpack loads are between 10 and 20% of their body weight. Lumbar and cervical spine loading is influenced by the backpack’s design and weight distribution [4], and backpack designs have changed to lessen spinal loading. Balance backpacks (BBP) are a new carriage system allowing a user to balance loads evenly and claim to reduce a forward leaning gait [5]. Traditional backpacks (TBP) have posterior load carriage, whereas a BBP claims to balance the loading and reduce posterior loading [6]. Balanced backpack load placement [7] may encourage lower energy expenditure and delay fatigue onset [8,9]. Heavy posterior backpack loading causes a forward leaning gait [6,7,8,9,10,11], with significant postural changes observed when backpack loads reach 10% of the user’s body weight (BW) [12]. To maintain postural balance, a decrease in maximum lumbar extension (LE), or a forward lean, occurs to compensate for the heavy posterior loading. The trunk aligns optimally over the hip to minimise hip flexion and to improve gait efficiency [13]. This adaptation of trunk angle has been observed among soldiers and hikers while carrying heavy equipment [14,15]. Posterior loading shifts the line of gravity before the base of support, leading to trunk flexion to restore stability and increased LE to maintain balance, causing higher spinal muscle recruitment, and leading to fatigue and discomfort [11].

Heavy backpack loads are associated with an observed increase in the applied ground reaction forces (GRF) during gait, as the musculoskeletal system compensates to maintain dynamic stability under additional mechanical demands [12,13,16]. The centre of pressure (COP), which is the point location of the vertical GRF vector, serves as a critical biomechanical indicator of postural control and reflects the neuromuscular system’s ability to regulate the body’s centre of mass (COM) over the base of support during locomotion [17,18]. While COP metrics such as average displacement, maximum excursion, and range do not directly quantify dynamic stability, they still offer indirect yet sensitive measures of gait control, particularly in response to external loading [17]. Variations in COP trajectory, especially increased range and variability, have been interpreted as compensatory responses aimed at preserving balance and minimising fall risk under loaded conditions [17,19].

Furthermore, both the magnitude and placement of anterior–posterior external loads significantly influence COP and trunk kinematics. Load carriage has been shown to alter anterior–posterior COP displacement even at relatively low load thresholds equal to 5–10% of body weight. This displacement could potentially disrupt the smooth progression of the COM and increase energy costs during gait [20,21,22]. Because COP displacement reflects the dynamic interplay between mechanical loading, neuromuscular coordination, and movement efficiency, it serves as a useful proxy for assessing gait adaptations during load carriage [22,23].

Given the biomechanical linkage between COP, LE and trunk kinematics during backpack carriage and gait, the present study aimed to evaluate LE and COP changes under three carriage conditions, no backpack (NBP), a traditional backpack (TBP), and a balanced backpack (BBP), when walking on flat, incline, and decline gradients. The primary aim was to observe LE and COP changes while walking on a flat treadmill under three loading conditions (NBP, TBP, and BBP). The secondary aim was to observe the LE and COP changes while walking on an incline/decline treadmill under three loading conditions (NBP, TBP, and BBP).

## 2. Materials and Methods

### 2.1. Study Design

The University of Canterbury’s Human Research Ethics Committee (Ref: 2023/104/LR) approved the research. Eight participants (age, 23 ± 2 years; weight, 85 ± 8 kg; height, 1.85 ± 0.04 m) provided voluntary written consent to participate in the study and met the following criteria: (1) must have taken at least one overnight hike in the last 12 months; (2) must have experience in carrying hiking backpacks; (3) must be aged between 18 and 40 years. Participants under medication or having any medical condition at the time of the study were excluded.

### 2.2. Equipment

Two different backpack (TBP and BBP) designs were tested; the backpack brand names and their images are confidential to comply with the research ethics agreement. The backpacks were fitted following each manufacturer’s guidelines with the shoulder, sternum, and hip straps adjusted to each participant’s torso dimensions. Both the TBP and BBP were custom loaded to 15% of each participant’s BW (12.5 ± 1.5 kg) using sandbags and weight plates [24]. The BBP and TBP load distribution was spread evenly and balanced bilaterally [25]. In addition, the BBP’s front packs were also loaded, with an anterior–posterior load ratio of 3:7. Three-dimensional gait kinematic data and kinetic data were collected using a 12-camera Optitrack motion capture system, Motive (2.2.0 (48012)), and AMTI Treadmetrix force instrumented treadmill (XCIE6); sampling was carried out at 240 Hz and 2000 Hz. The Gait2392, Plug In-Gait model, OpenSim (OpenSim 4.1, Stanford, CA, USA), was used as an inverse kinematic software to assess LE and COP during backpack carriage. The treadmill’s embedded force plates provided COP coordinates to assess the COP displacement values.

### 2.3. Procedure

After anthropometric assessments, a test and equipment familiarisation session were provided for each participant including a treadmill walking warmup. During the warmup, equipment familiarisation, and treadmill testing, the participants were advised to wear their personal hiking shoes. Reflective markers (*n* = 29) on the participant’s Velcro suits were placed bilaterally on the acromion process, anterior superior iliac spine, sacrum, lateral femoral condyle, and lateral malleolus. Participants were given a verbal command “walk normally, swinging your arms with a posture and pace that is comfortable to maintain” to enable them walk on the treadmill using their preferred walking pattern.

To simulate outdoor walking, three gradients were selected in a repeat-measures experiment design: 0° (flat), 12° (incline), and −12° (decline). The instrumented treadmill was set at a consistent walking speed of 1.1 m/s [26]. During each trial, each gradient was first set to flat, then incline, and then decline. Each backpack walking variation lasted two minutes. The gradient was then adjusted and repeated to record the respective gradient condition variables. Participants first completed the trial with no backpack (NBP) under the flat condition so that each subject’s normal gait data could be captured without any influencing variables [27]. Then, the trials carrying either the TBP or BBP were randomly assigned, and data were collected for each surface gradient, with a five-minute rest between each trial.

### 2.4. Data Collection

**Figure 1:**
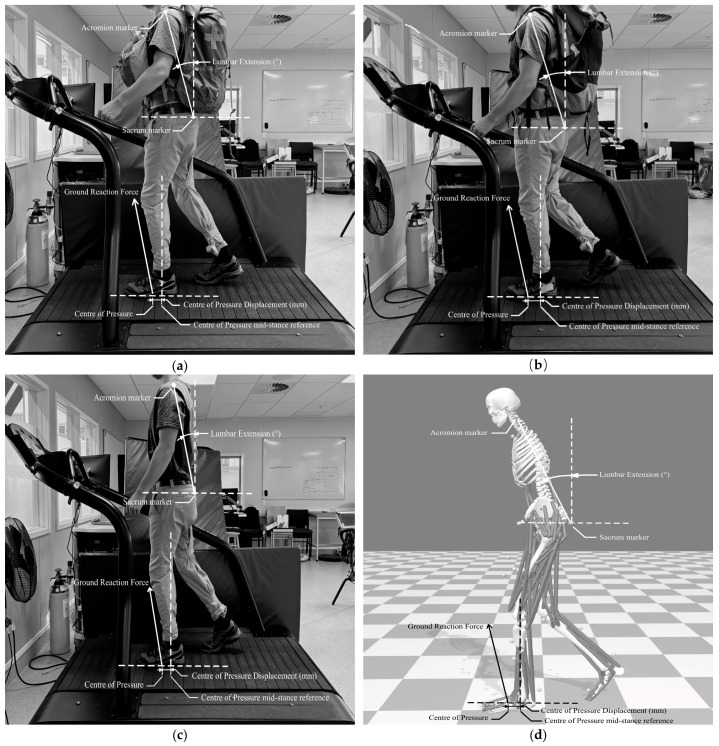
(**a**) An illustration showing the balanced backpack condition. (**b**) An illustration showing the traditional backpack condition. (**c**) An illustration showing the no-backpack condition. (**d**) An illustration to visualise positive and negative lumbar extension and the centre of pressure displacement range.

Treadmill embedded force plate and motion capture data were organised using Microsoft Excel. Each step was distinguished between time stamps of consecutive peak vertical forces, which was the initial contact of the leading foot during double support [28,29]. An illustration of how LE was measured is provided in Figure 1. The LE angle was measured from a line perpendicular to the ground, with a forward lean resulting in a negative extension value (decrease in LE). The LE average was measured as the average lumbar angle during the specific step cycle. The LE maximum was measured as the largest forward lean (negative value) angle during the specific step cycle. The LE ROM was measured as the difference between the maximum and minimum LE angles during the specific step cycle. Similarly, the COP value was measured along the x-axis (anterior and posterior) from the calculated centre pressure point of the participant’s foot, with positive COP displacement values being placed further forward and negative further behind. The COP displacement average was measured as the average displacement distance during the specific step cycle. The COP displacement maximum was calculated as the largest displacement distance during a specific step cycle. The COP displacement range refers to the maximum distance the COP travels from a reference point in the anterior–posterior direction. The reference point was COP location during mid-stance. Mid-stance was defined as the moment where the ground reaction force was closest to perpendicular in relation to the treadmill surface. In this study, the COP ROM was calculated as the difference between the maximum and minimum displacement distance during a specific step cycle. An illustration of how COP was measured is provided in Figure 1. For each gradient and backpack condition, participants completed a 60 s acclimatisation period on the treadmill to adapt to the gait pattern. Data were subsequently collected during continuous walking, with only the post- acclimatisation period data used for analysis. The average LE ROM and COP displacement data were recorded and averaged for each step.

### 2.5. Statistical Analysis

All statistical analyses were performed on Prism (Version 10.1.1, GraphPad Software, LLC). Variable data distribution normality was assessed and verified using a Shapiro–Wilk test, indicating no significant data deviation from normality (*p* < 0.05). The LE and COP variables were analysed using a repeated-measures Anova (IBM SPSS Statistics Version 29) design to contrast the TBP and BBP with NBP across the varying gradient conditions [30,31,32]. Effect size was reported using partial eta squared (η^2^), with values closer to one indicating a greater proportion of total variance. A “high” effect size was considered when η^2^ > 0.14; a “medium” effect size was considered when η^2^ > 0.06; and a “low” effect size was considered when η^2^ > 0.01 [30,31,32]. When significant effect levels were recognised, Dunnett’s multiple comparison test was utilised to differentiate the effect that each backpack configuration had on the variable of interest. Type I error risk was reduced with Dunnett’s comparison correction (with a set alpha level of 0.05) [30,31,32]. Geisser–Greenhouse’s Epsilon correction was employed when data sphericity was violated.

## 3. Results

### 3.1. Lumbar Extension

When tested under different gradients, the mean differences in the average LE ROM between NBP and TBP were 4.74° (flat), 3.75° (incline), and 5.68° (decline); for the maximum LE ROM, the differences were 4.64° (flat), 3.56° (incline), and 4.95° (decline), as shown in Table 1.

In comparison, differences between NBP and BBP were smaller, 0.67° (flat), 0.23° (incline), and 1.23° (decline), for the average LE ROM, and 0.87° (flat), 0.96° (incline), and 1.04° (decline) for the maximum LE ROM. Backpack type had a significant main effect (*p* < 0.001) on both average and maximum LE values, as shown in Table 2. Mean LE data indicated a consistent forward lean across all conditions, with more pronounced extension observed when using the TBP. The effect size of the backpack condition was high for both average and maximum LE values, accounting for 39% (η^2^ = 0.385) and 30% (η^2^ = 0.300) of the total difference, respectively. In contrast, its effect on the LE ROM was low (η^2^ = 0.005).

Dunnett’s post hoc test revealed significant differences (*p* < 0.05) with the TBP for both average and maximum LE values, while no significant differences were observed with the BBP, indicating that the TBP was the primary contributor to the changes in LE, as displayed in Table 3. Gradient variation significantly affected the average and ROM means of LE, both with high effect sizes (η^2^ > 0.15). Interaction effects between backpack and gradient for the LE average, maximum, and ROM values were all in the medium-to-low range (η^2^ < 0.15).

### 3.2. Centre of Pressure

Backpack type had a significant main effect on average COP displacement (*p* < 0.001), with a high effect size (η^2^ = 0.021), while maximum displacement (*p* = 0.070) and ROM (*p* = 0.265) showed a medium-to-low effect size. Dunnett’s post hoc test indicated significant differences between the TBP and NBP (*p* < 0.05), but not between the BBP and NBP (*p* < 0.05), as displayed in Table 3. Surface gradient had a strong effect on all COP variables, with high effect sizes for average (η^2^ = 0.839; *p* < 0.001), maximum (η^2^ = 0.579; *p* < 0.001), and ROM (η^2^ = 0.355; *p* = 0.008) displacement. Backpack x gradient interaction effects were high for average (*p* = 0.004) and maximum COP displacement (*p* = 0.010) but low for the COP ROM. Adjusted *p*-values for average COP displacement across gradients confirmed this interaction (flat, *p* = 0.002; incline, *p* = 0.028; decline, *p* = 0.030). When using the TBP, average COP displacement increased by 16.2 mm for both flat and incline gradients (anterior to the COP) and decreased for the decline gradient (posterior to the COP). No significant differences were observed across backpack conditions for the COP displacement maximum or ROM, as shown in Table 1. Mean differences in average COP displacement between the NBP and TBP were 15.8 mm (flat), 7.6 mm (incline), and 4.7 mm (decline); for maximum COP displacement, the differences were 7.7 mm (flat), 12.8 mm (incline), and −5.1 mm (decline). In contrast, the BBP showed smaller differences from the NBP: 1.4 mm (flat), 3.9 mm (incline), and −1.0 mm (decline) for average COP displacement, and −1.0 mm (flat), 9.5 mm (incline), and −2.2 mm (decline) for maximum COP displacement.

## 4. Discussion

This study examined LE and COP displacement in hikers carrying different backpack types while walking on two gradients. The primary aim was to observe LE and COP changes while walking on a flat treadmill under three loading conditions (NBP, TBP, and BBP). A forward-tilted LE posture was observed during TBP and BBP use on flat treadmill walking, consistent with expected postural adaptations to load carriage. The secondary aim was to observe the LE and COP changes while walking on an incline/decline treadmill under three loading conditions (NBP, TBP, and BBP). These trials revealed that the forward-leaning posture persisted across gradients and did not diminish during decline gradient walking. Notably, BBP use resulted in significantly lower COP displacement compared to TBP use, suggesting that its more balanced load distribution may enhance postural stability across different walking gradients.

### 4.1. Lumbar Extension

Across all gradients, the TBP produced significantly greater reductions in both average and maximum LE values compared to the NBP, with the largest differences observed during the decline condition, followed by the flat and incline conditions. In contrast, differences between the NBP and BBP were minimal and not statistically significant, consistent with prior findings that posteriorly distributed loads result in less trunk angle deviation. This aligns with studies reporting smaller trunk angle changes when using non-traditional loading systems (lateral loading) compared to traditional anterior loads [6]. The BBP’s 30/70 anterior–posterior weight distribution likely shifted the centre of gravity forward, closer to its natural vertical alignment over the base of support, thereby promoting a more upright posture. This adjustment is consistent with gait research showing that backpack carriage exceeding 15% of body weight significantly affects trunk posture [25,33]. Given the functional demands of hiking, maintaining efficient gait mechanics is essential as a prolonged forward lean can lead to trunk muscle fatigue and strain.

High recruitment of the rectus abdominis during heavy carriage has been observed as a counterbalance response to additional posterior load [24]. Our results indicate that during BBP gait carriage, the lumbar position resembles an unloaded gait. Previous research has indicated LE ROM changes with insignificant effect sizes when considering a step’s LE ROM during backpack carriage [34,35,36]. In our study, the LE ROM remained unaffected by backpack variation, which could be attributed to the discrepancy between studies when distributing the posterior mass. We ensured an evenly distributed load through the pack’s internal compartment and specifically packed the mass closer to the user’s back, extending uniformly from the shoulders to the hips, thus minimising angular momentum compared to traditional backpacks.

### 4.2. Centre of Pressure

During every gait cycle, as weight transitions from the heel to the toe, the average displacement from the individual foot’s centre pressure point determines whether the participant’s weight is shifting forward or backward. Mean value trends, displayed in Table 1, provide less obvious changes in COP. Further examination of the significant effect of backpack variation on COP displacement revealed a greater data variance with the TBP than the BBP, suggesting that the conventional posterior loading system demands more adaptive measures to preserve gait balance. While the anterior–posterior loading system naturally provides a more balanced distribution of load, the mean difference in COP displacement average, when comparing NBP to TBP use, was the largest on flat conditions and similar during the incline and decline conditions. This aligns with a meta-analysis finding on postural sway during loading where two factors were highlighted as significant influences on postural stability: (1) COP anterior–posterior sway was increased forward during carriage with larger loads and (2) COP anterior–posterior sway was increased further forward during posterior load placements compared to either balanced or anterior load placement [20]. The body’s mechanism to adapt to changes in its mass inertia characteristics often explains postural sway, which has been reported to be present even during static standing while holding an additional mass [37]. The TBP creates unnatural body mass inertia, requiring biomechanical adjustment to maintain a stable gait. Furthermore, with the observed increase in vertical ground reaction forces during backpack carriage [16], a forward shift in the COP implies that the body will absorb the vertical ground reaction forces through an unnatural line of impact. Gait with an excessive forward lean increases angular impulse in hip extensors and knee flexors and decreases it in the hip flexors and knee extensors [27]. This increases the risk of musculoskeletal injuries as the system becomes fatigued and less resilient to impact forces [38,39].

It is also interesting to note the significant interaction between backpack and gradient, where the average COP displacement mean difference was less significant for both incline and decline between backpack variations. The influence of the TBP on the average COP was reduced during sloped conditions, with diminished forward weight displacement. Uphill gait adaptations typically involve adjustments in the thorax, hip, knee, and ankle, while downhill adaptations are more highly centred around the knee and ankle. It has been suggested that increased musculoskeletal activity and cognitive engagement result in reduced COP motion [20], providing an explanation of why our results demonstrated a reduced COP ROM during sloped gradients. The lab environment allows for accurate movement analysis but sacrifices an accurate representation of real-life hiking conditions. Each backpack condition’s effect on postural variables over a prolonged period of walking on uneven surfaces was limited to the acute changes observed on a consistently even treadmill belt.

## 5. Limitations

Recruiting experienced male hikers with similar stature and torso length proved logistically difficult, as it was necessary to minimise variability in torso length and ensure use of the same size backpacks. This limitation contributed to a low number of participants and is acknowledged as a constraint in this study. The controlled laboratory setting enabled precise gait measurements, but it lacked ecological validity. The effects of each backpack condition were limited to acute postural changes during treadmill walking, without accounting for prolonged exposure on uneven terrain typical of real-world hiking.

## 6. Conclusions

By investigating the differences between the TBP and BBP loading designs, we determined that the BBP’s anterior–posterior loading system more closely replicated the unloaded gait pattern in comparison to the TBP. Based on the lab simulation, our data suggest that hikers walking long distances carrying a balanced backpack may maintain a posture closely resembling their unloaded gait.

## Figures and Tables

**Table 1:** Mean (SD) of lumbar extension and centre of pressure variables.

Variable and Gradient		Backpack Type	
	NBP	TBP	BBP
Average LE (^o^)			
Flat	−11.56 ± (2.10)	−16.30 ± (2.32)	−12.23 ± (2.32)
Incline	−13.91 ± (1.89)	−17.66 ± (2.37)	−14.20 ± (2.53)
Decline	−9.64 ± (2.27)	−15.32 ± (2.97)	−10.88 ± (2.51)
Maximum LE (^o^)			
Flat	−13.06 ± (2.26)	−17.70 ± (2.54)	−13.92 ± (2.90)
Incline	−15.59 ± (2.08)	−19.15 ± (2.66)	−16.54 ± (3.33)
Decline	−12.03 ± (2.76)	−15.79 ± (3.04)	−13.07 ± (2.91)
LE ROM (^o^)			
Flat	2.51 ± (0.38)	2.36 ± (0.54)	2.28 ± (0.51)
Incline	2.72 ± (0.89)	2.98 ± (0.86)	3.09 ± (0.93)
Decline	4.04 ± (0.89)	3.68 ± (1.44)	3.41 ± (1.06)
Average COP displacement (mm)			
Flat	13.32 ± (7.31)	29.90 ± (8.21)	14.73 ± (8.28)
Incline	35.31 ± (11.02)	41.92 ± (15.02)	37.89 ± (15.83)
Decline	−25.68 ± (9.22)	−22.22 ± (11.84)	−27.87 ± (6.45)
Maximum COP displacement (mm)			
Flat	103.36 ± (3.51)	111.31 ± (13.51)	102.82 ± (9.62)
Incline	124.40 ± (14.43)	137.62 ± (16.17)	133.31 ± (15.22)
Decline	92.32 ± (19.98)	87.63 ± (14.69)	90.11 ± (21.01)
COP ROM (mm)			
Flat	269.89 ± (23.73)	265.99 ± (24.96)	262.82 ± (12.34)
Incline	292.39 ± (34.70)	293.53 ± (26.53)	295.93 ± (29.48)
Decline	254.54 ± (34.22)	241.06 ± (32.14)	244.45 ± (29.34)

Note: NBP, no backpack; TBP, traditional backpack; BBP, balance backpack; COP, centre of pressure; ROM, range of motion; LE, lumbar extension.

**Table 2:** ANOVA analysis summary of backpack, gradient, and interaction.

Variable	Condition	F (*p*)	Significance (*p* < 0.05)	Effect Size (η^2^)
Average LE (^o^)	Backpack loading	192.9 (<0.001)	Yes	0.385
	Treadmill gradient	4.0 (0.034)	Yes	0.162
Maximum LE (^o^)	Backpack loading	116.0 (<0.001)	Yes	0.300
LE ROM (^o^)	Backpack loading	0.4 (0.626)	No	0.005
	Treadmill gradient	7.3 (<0.001)	Yes	0.554
Average COP displacement (mm)	Backpack loading	28.4 (<0.001)	Yes	0.021
	Treadmill gradient	69.7 (<0.001)	Yes	0.839
	Interaction	4.4 (0.004)	Yes	0.006
Maximum COP displacement (mm)	Backpack loading	2.9 (0.070)	No	0.009
	Treadmill gradient	17.8 (<0.001)	Yes	0.579
	Interaction	3.8 (0.010)	Yes	0.024
COP ROM (mm)	Backpack loading	1.4 (0.265)	No	0.005
	Treadmill gradient	6.3 (0.008)	Yes	0.355

Note: LE, lumbar Extension; ROM, range of motion; COP, centre of pressure; F, F-statistics; η^2^, eta-squared.

**Table 3:** Post hoc comparison of significant backpack effects.

Variable	Mean Difference		Adjusted *p* Value	
	TBP	BBP	TBP	BBP
Average LE (^o^)				
Flat	4.741	0.6687	<0.001	0.374
Incline	3.746	0.2888	<0.001	0.622
Decline	5.679	1.233	<0.001	0.054
Maximum LE (^o^)				
Level	4.64	0.866	<0.001	0.262
Incline	3.56	0.956	<0.001	0.359
Decline	4.95	1.233	<0.001	0.223
Average COP displacement (mm)				
Level	−1.58	−0.138	0.002	0.491
Incline	−0.763	−0.388	0.028	0.148
Decline	−0.471	0.1	0.030	0.855

Note: TBP, traditional backpack; BBP, balance backpack; LE, lumbar extension; COP, centre of pressure.

## Data Availability

The data that supports the findings of this study are available on request.

## Abbreviations

The following abbreviations are used in this manuscript:

COP	Centre of pressure
LE	Lumbar extension
BBP	Balance backpack
NBP	No backpack
TBP	Traditional backpack
